# Adaptation of the Clinical Dementia Rating Scale for adults with Down syndrome

**DOI:** 10.1186/s11689-019-9300-2

**Published:** 2019-12-16

**Authors:** Christina N. Lessov-Schlaggar, Olga L. del Rosario, John C. Morris, Beau M. Ances, Bradley L. Schlaggar, John N. Constantino

**Affiliations:** 1 Department of Psychiatry, Washington University School of Medicine, St. Louis, MO USA; 2 Intellectual and Developmental Disabilities Research Center, Washington University School of Medicine, St. Louis, MO USA; 3 Department of Neurology, Washington University School of Medicine, St. Louis, MO USA; 4 Knight Alzheimer Disease Research Center, Washington University School of Medicine, St. Louis, MO USA; 5 Department of Radiology, Washington University School of Medicine, St. Louis, MO USA; 6 Department of Neuroscience, Washington University School of Medicine, St. Louis, MO USA; 7 Kennedy Krieger Institute, Baltimore, MD USA; 8 Department of Neurology, Johns Hopkins University School of Medicine, Baltimore, MD USA; 9 Department of Pediatrics, Johns Hopkins University School of Medicine, Baltimore, MD USA

**Keywords:** Down syndrome, Dementia, Premorbid ability, Cognitive decline, Cognitive impairment

## Abstract

**Background:**

Adults with Down syndrome (DS) are at increased risk for Alzheimer disease dementia, and there is a pressing need for the development of assessment instruments that differentiate chronic cognitive impairment, acute neuropsychiatric symptomatology, and dementia in this population of patients.

**Methods:**

We adapted a widely used instrument, the Clinical Dementia Rating (CDR) Scale, which is a component of the Uniform Data Set used by all federally funded Alzheimer Disease Centers for use in adults with DS, and tested the instrument among 34 DS patients recruited from the community. The participants were assessed using two versions of the modified CDR—a caregiver questionnaire and an in-person interview involving both the caregiver and the DS adult. Assessment also included the Dementia Scale for Down Syndrome (DSDS) and the Raven’s Progressive Matrices to estimate IQ.

**Results:**

Both modified questionnaire and interview instruments captured a range of cognitive impairments, a majority of which were found to be chronic when accounting for premorbid function. Two individuals in the sample were strongly suspected to have early dementia, both of whom had elevated scores on the modified CDR instruments. Among individuals rated as having no dementia based on the DSDS, about half showed subthreshold impairments on the modified CDR instruments; there was substantial agreement between caregiver questionnaire screening and in-person interview of caregivers and DS adults.

**Conclusions:**

The modified questionnaire and interview instruments capture a range of impairment in DS adults, including subthreshold symptomatology, and the instruments provide complementary information relevant to the ascertainment of dementia in DS. Decline was seen across all cognitive domains and was generally positively related to age and negatively related to IQ. Most importantly, adjusting instrument scores for chronic, premorbid impairment drastically shifted the distribution toward lower (no impairment) scores.

## Background

Alzheimer disease (AD) is the most common cause of dementia [1]. Because of the aging population worldwide, AD is reaching epidemic proportions; in 2018, an estimated 5.5 million United States citizens age 65 and older had AD, which is projected to grow to 13.8 million by the middle of this century if no effective therapies are developed to prevent, slow, or stop the disease [2]. Down syndrome (DS) is the most commonly identified genetic cause of cognitive impairment. Adults with DS have an increased risk for developing AD dementia owing to the trisomy of chromosome 21 where the amyloid precursor protein (*APP*) gene is located leading to overexpression of both APP mRNA and amyloid beta (Aβ) peptide [3]. Dementia is often identified earlier in adults with DS compared with the typical aging population, with estimates of about 10% in DS adults in their 40s, about 30% in their 50s, and as high as 80% of DS adults in their 60s [3–5].

Cognitive and functional decline are the salient indicators of AD [2, 6]. DS adults have pre-existing and varying levels of intellectual disability and cognitive impairment [7] making it difficult to assess change relative to a typical level of functioning. In the DS population, assessment of change in cognitive function must take into account baseline cognitive abilities [8, 9]. One of the first instruments to be modified for use in DS was the Dementia Questionnaire for Persons with Mental Retardation (DMR [10, 11], since renamed the Dementia Questionnaire for People with Learning Disabilities (DLD)). Criticisms of the DMR/DLD include lack of assessment of change from earlier level of functioning, which results in significant floor effects, and use of different cutoff scores of increasing stringency for higher levels of intellectual disability, the latter of which is not always known or possible to assess [12–14].

The Dementia Scale for Down Syndrome (DSDS) [15] is an informant interview specifically for use in DS. It contains a total of 60 items scored as present, absent, typical of the individual, or not applicable; 20 items assess early-stage dementia, 20 items assess middle stage dementia, 15 items assess late-stage dementia, and 5 items assess very late-stage dementia [15]. The DSDS was developed and standardized in a sample of adults the majority of whom had severe to profound intellectual disability; hence, the most common criticism is that it may not be sensitive enough to detect dementia in higher functioning individuals [16, 17]. A counterargument to this criticism is that informants may not be able to detect early dementia-related changes in such individuals [17].

The Cambridge Examination for Mental Disorders in the Elderly (CAMDEX) [18] is an informant interview developed for neuropsychiatric diagnosis in the elderly, including dementia. It was modified for use in DS (CAMDEX-DS) [19, 20]. The CAMDEX-DS consists of approximately 150 questions that ask about functional difficulties in different cognitive domains, whether such difficulties represent a deterioration in function, and the extent of the deterioration (slight or great) [20]. The CAMDEX-DS has moderate to high inter-rater reliability, good predictive validity, and correlates with change in independent cognitive tasks administered to DS adults longitudinally [19].

The Dementia Screening Questionnaire for Individuals with Intellectual Disabilities (DSQIID [12]) was more recently developed and is gaining wider use and popularity. The DSQIID focuses specifically on change in function relative to a premorbid level; it is brief and easy to complete by a single informant. It has been translated into multiple languages including Chinese [21] and Italian [13], and along with the original English version, all versions have good psychometric properties [12, 13, 21].

In addition to these advances, it would be desirable to adapt clinical staging instruments that are widely utilized in scientific and clinical work involving AD dementia in the broader population to afford comparability of assessment and staging for scientific and clinical efforts involving individuals with DS. Therefore, we adapted the Clinical Dementia Rating scale (CDR [22]) for use in DS. The CDR is a semi-structured clinical diagnostic interview that is an integral component of the Uniform Data Set [23] that is used by all federally funded Alzheimer Disease Centers (ADC) to determine the presence or absence of dementia for individuals being evaluated at ADCs. The semi-structured interviews are administered separately to an informant and then to the research participant or patient and focus on capturing intra-individual change from previous cognitive and functional performance levels. An experienced clinician then synthesizes the information from the interview to determine the presence or absence of dementia and, when present, its severity [22]. A CDR score of 0 indicates cognitive normality, whereas scores of 0.5, 1, 2, and 3 indicate very mild, mild, moderate, and severe dementia, respectively.

The primary goal of this project was to determine whether adaptations of the CDR for DS—by both interview and caregiver report—could reliably capture variation among DS patients when implementing new adaptations of an established dementia rating scale.

## Methods

### Sample

In the first study phase, families with DS adults aged 18 years and older were recruited from the St. Louis ARC, a non-profit organization that provides support and services for individuals with intellectual and developmental disabilities in the St. Louis region. Potential participants were recruited via letters sent to the families by St. Louis ARC. A total of 189 letters were sent asking families to contact the study. Forty families (21.2% response rate) contacted study staff over the telephone regarding their interest to participate. Verbal consent was obtained from the caregiver over the telephone and he/she was then sent the questionnaire version of the modified CDR instrument (CDR-QDS) by postal mail. All forty CDR-QDS instruments that were mailed were received back for evaluation.

In the second study phase, families who completed the CDR-QDS were subsequently invited to come to the laboratory. Families who agreed to participate after verbal explanation over the telephone of this study phase were sent the informed consent document to their homes prior to their laboratory appointment. At the time of the appointment, study staff went over study procedures, answered any questions, and obtained signed informed consent from the caregiver/legal guardian and signed ascent from the DS adult. A question as to whether the DS adult knew why he/she is in the laboratory was asked to both the informant and the DS adult as part of the interview process, to assess capacity.

The laboratory appointment involved administration of (1) the modified interview format of the CDR (CDR-IDS) to the informant and to the DS adult; (2) the Dementia Scale for Down Syndrome (DSDS) [15], for convergent validity against which scores on the modified CDR-QDS and CDR-IDS instruments were to be compared. The DSDS informant interview is used as a screening tool for dementia diagnosis in the multisite, longitudinal Neurodegeneration in Aging Down Syndrome study (NiAD) [24, 25]. It has high inter-rater reliability [15], moderate to high specificity and sensitivity compared with clinical dementia diagnosis using DSM-IV [26] or ICD-10 criteria [27], it correlates positively with DMR scores [16, 28, 29] and is related to worse memory task performance [29, 30] and lower scores on information and orientation questions [30]; and (3) the Raven’s Colored Progressive Matrices task [31] administered to the DS adult to estimate intelligence quotient (IQ). This task was chosen as a reasonable estimate of IQ that would not be limited by speech impairment that can be observed in DS adults.

Results from this study were not given to patient families. This study was approved by the Institutional Review Board at Washington University in St. Louis.

### Instrument modification

#### Questionnaire version: CDR-QDS

Original CDR questions were uniformly modified to ascertain degree of cognitive impairment and the extent to which change had occurred in relation to the participant’s prior baseline. In order to effect this general modification, in some cases, we generated qualifiers for affirmative responses; for example, endorsement of the CDR question “Does he/she have problems with his/her memory or thinking?” triggered a new follow-up question: “Did he/she ALWAYS have a problem with his/her memory or thinking?” (Yes (1)/No (0)). If the DS adult was reported to currently have problems with memory or thinking (current score = 1) but he/she always had this problem (always score = 1), then the CDR-DS rating for cognitive decline was calculated to be zero (current score 1–always score 1 = 0).

In other cases, items related to the DS adult’s *current level of functioning* were paired with identical companion items eliciting an endorsement of the same item with respect to his/her *best ever level of functioning*; for example, the CDR question “How often does he/she know the exact day of the month?” has response categories: Usually (0), Sometimes (1), Rarely (2), and Don’t Know. If the DS adult was reported to *currently* Rarely know the exact day of the month but his/her *best ever* ability was that he/she Sometimes knew the exact day of the month, then the decline score would be 1 (current score 2–best ever score 1).

In still other cases, we changed response categories; for example, the original CDR question “Rate his/her ability to cope with small sums of money (e.g., calculate change, leave a small tip)” has response categories: No Loss (0), Some Loss (1), and Severe Loss (2). We substituted No Loss with Never Able (0) and Some Ability/No Loss (0) response categories and kept Some Loss (1) and Severe Loss (2). A difference (i.e., adjusted) score could not be calculated for questions with these response options since change relative to premorbid ability was implicit in the response options.

CDR-QDS scores were generated for each of six functional domains of the CDR—memory, judgment and problem solving, orientation, community affairs, home and hobbies, and personal care—by summing over response categories of individual questions/items within each domain. Higher scores reflected greater cognitive decline. Cognitive domain scores were summed for a total CDR-QDS summary score. Unlike the interview instrument described below, we did not generate CDR-QDS global scores because published rules for generating a global score [22] are based on interview data with both an informant and the participant and include clinical judgment garnered through direct observation and personal interaction with the informant and patient.

#### Interview version: CDR-IDS

Examination of CDR-QDS data prompted modifications that were included in the CDR-IDS. In addition, unlike the CDR-QDS where a cognitive decline score was generated for every item, CDR-IDS scoring included clinical judgment as well as direct assessment of the DS adult. Two members of the team (CNLS and OLDR) underwent CDR clinical training through the Knight Alzheimer Disease Research Center at Washington University. One member (OLDR) performed the face-to-face interviews with the informant and the DS adult and the other reviewed the video recordings of the interviews. We each scored the interviews separately and together reached a consensus on disparate scores by discussing rationales for the original scores. Consistent with CDR scoring [22], each cognitive domain was given a score of 0 (typical), and 0.5, 1, 2, or 3 reflecting very mild, mild, moderate, or severe dementia respectively. A sum of boxes CDR-IDS score was generated by summing across cognitive domain scores as well as a global CDR-IDS score generated using published rules that weighted the memory domain score more heavily than other domains [22]. The extended number of modified questions in the CDR-IDS along with the difference in the scoring process allowed us to generate scores that reflected no adjustment (i.e., current scores) or adjustment for premorbid ability.

### Statistical analysis

Data were managed using REDCap electronic data capture tools [32] hosted by the Washington University School of Medicine. REDCap (Research Electronic Data Capture) is a secure, web-based application designed to support data capture for research studies, providing (1) an intuitive interface for validated data entry; (2) audit trails for tracking data manipulation and export procedures; (3) automated export procedures for seamless data downloads to common statistical packages; and (4) procedures for importing data from external sources. Similarity in cognitive decline scores across cognitive domains and across modified questionnaire and interview instruments was estimated using Spearman rank order correlation. Spearman correlation was also used to examine the association of cognitive decline scores with age and IQ. Inter-rater reliability in scoring of the modified CDR-IDS interview was estimated using the kappa coefficient [33].

## Results

### Sample characteristics

Of the 40 returned CDR-QDS questionnaires, 6 were incomplete due to missing data. For the remaining 34 completed questionnaires (2 DS adults were nonverbal), mean age of the DS adults was 33.2 years old (SD = 9.7; range 18–55 years), 15 were women (44.1%), 30 DS adults lived with the responder (88.2%), and the responder was the mother for 28 of the DS adults (82.4%), with the father and full sibling reporting on 4 and 2 of the DS adults, respectively (11.8% and 5.9%). Respondents reported spending an average of 55.5 h per week with the DS adult (SD = 29.5, range 4–112 h).

Thirty-three of the 34 families with CDR-QDS data were invited to come to the laboratory. From these 33 families, 22 completed assessments in the laboratory. Of the 11 families who did not come to the laboratory, 5 declined to participate, 4 withdrew from the study after initially agreeing to participate, and 2 families did not respond to repeated calls. Five of the 9 families who declined to participate or withdrew from the study had reported relatively high CDR-QDS scores for the DS adult (≥ 14 where the scoring range was 0–32). The 22 participant families comprised 10 women with DS (45.5%), all non-Hispanic White, of average age 32.8 (SD = 8.9, range 20–56 years) and average IQ of 59 (SD = 19.5, range 24–85; IQ was not computed for two individuals who were not able to complete Raven’s task, one of whom was nonverbal).

History of medical and behavioral problems was obtained from the differential diagnosis screening questions of the DSDS [15]. Of the 22 DS adults, 16 (72.7%) had hypothyroidism; 9 (40.9%) had hearing/ear problems; 5 (22.7%) had vision problems; 5 had heart problems; 6 (27.3%) had sleep apnea; 5 had depression, anxiety, or mood problems; 2 (9.1%) had suicidal ideation; 11 (50%) had obsessive-compulsive disorder; and 2 had aggressive behavior including verbal aggression.

There was 80% power to detect a correlation coefficient ≥ |0.44| (2-tailed alpha) with *n* = 34 and a coefficient ≥ |0.53| (2-tailed alpha) with *n* = 22.

### CDR-IDS inter-rater reliability

Table 1 shows that inter-rater reliability was very high for CDR-IDS cognitive domain and global scores adjusted for premorbid function (kappas = 0.82 to 1). Inter-rater reliability was overall high for unadjusted CDR-IDS scores (kappas = 0.79 to 1) with the weakest reliability (kappa = 0.55) for the judgment and problem solving cognitive domain.

**Table 1:** Inter-rater reliability for CDR-IDS scores before (unadjusted) and after (adjusted) accounting for premorbid function

CDR-IDS domain and global scores *n* = 22	Unadjusted		Adjusted	
	Kappa	SE	Kappa	SE
Memory	0.79	0.11	0.91	0.08
Orientation	0.94	0.06	1.00	0.00
Judgment and problem solving	0.55	0.13	0.82	0.15
Community affairs	0.92	0.08	1.00	0.00
Home and hobbies	1.00	0.00	1.00	0.00
Personal care	0.86	0.09	1.00	0.00
CDR-IDS global score	0.92	0.08	0.92	0.08

### Distribution of CDR-QDS and CDR-IDS scores

**Fig. 1:**
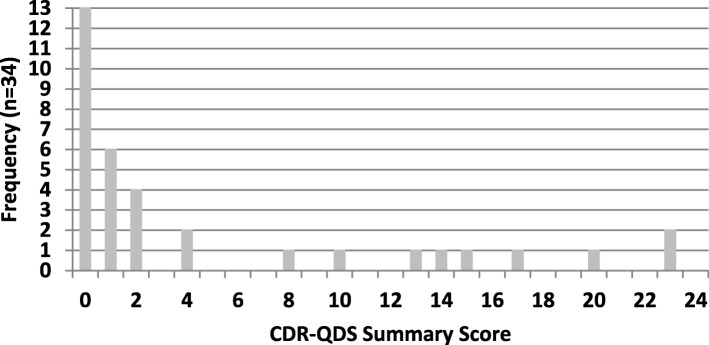
Frequency distribution of summary scores of the CDR questionnaire for Down syndrome (CDR-QDS)

Figure 1 shows the distribution of summary CDR-QDS scores (*n* = 34); the sample manifested a wide range of scores. The distribution was skewed toward lower scores where 13 individuals had a score of 0 (38.2%), and another 10 individuals had scores of 1 or 2 (29.4%). CDR-QDS summary score mean was 4.85 (SD = 7.29, median = 1, range 0–23, maximum possible score = 32). There were three individuals with CDR-QDS scores ≥ 20, all of whom were over the age of 30 years.

**Fig. 2:**
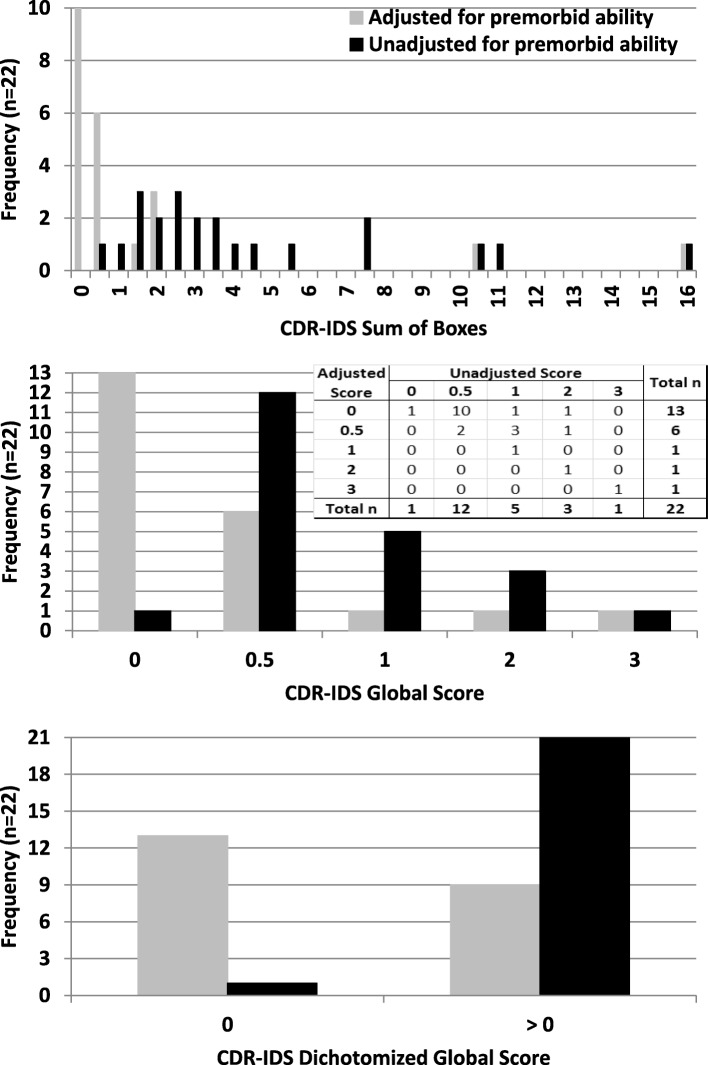
Frequency distributions of the CDR interview for Down *s*yndrome (CDR-IDS) sum of boxes (upper panel), global scores (middle panel), and dichotomized global scores (lower panel). Score distributions are shown before and after adjustment for premorbid ability. The table embedded in the middle panel illustrates the shift of individuals from higher unadjusted to lower adjusted global scores

Figure 2 shows the distribution of CDR-IDS sum of boxes (upper panel), global (middle panel), and dichotomized global scores (lower panel) (*n* = 22) when scores were adjusted for premorbid function (adjusted) and not adjusted (unadjusted), which would be consistent with *current* function. As predicted, adjusting for premorbid ability shifted, in the non-pathological direction, scores that would otherwise be elevated on the standard version of the CDR. CDR-IDS adjusted sum of boxes sample mean was 1.68 (SD = 3.90, median = 0.5, range 0–16, maximum possible score = 18) and unadjusted mean was 4.41 (SD = 3.88, median = 3, range 0.5–16). The table embedded in the middle panel of Fig. 2 shows the extent to which adjustment for premorbid ability shifted individuals to lower global CDR-IDS scores. For example, of the 13 DS adults with adjusted global CDR-IDS scores of 0, 10 had unadjusted scores of 0.5. The shift from any severity of dementia (CDR-IDS > 0) to no dementia (CDR-IDS = 0) is most pronounced in the lower panel, where nearly the entire sample (21 of 22 DS adults) meets criteria for at least very mild dementia before score adjustment, but only 9 individuals meet the dichotomized dementia threshold after score adjustment.

### Relationship of age and IQ to CDR-QDS and CDR-IDS

Table 2 shows that older age was significantly related to greater decline in all CDR-QDS cognitive domains, except for memory and total score. When individuals with zero scores were excluded from correlation estimation, older age was significantly related to both memory decline (Spearman rho = 0.49, *p* < 0.05, *n* = 17) and to total CDR-QDS score (rho = 0.41, *p* < 0.05, *n* = 21).

**Table 2:** Spearman correlations of cognitive domains and summary scores with age and IQ

Domains and summary scores	CDR-QDS (*n* = 34)		CDR-IDS (*n* = 22)	
	Age	IQ (*n* = 20)	Age	IQ (*n* = 20)
Memory	0.11	− 0.35	0.21	− 0.32
Orientation	*0.37*	− *0.47*	0.13	− 0.27
Judgment and problem solving	*0.40*	− 0.42	0.31	− 0.36
Community affairs	*0.35*	− *0.62*	0.38	− 0.30
Home and hobbies	*0.36*	− *0.49*	0.13	− 0.41
Personal care	*0.37*	− *0.49*	0.39	− *0.54*
CDR-QDS summary/CDR-IDS sum of boxes	0.11	− *0.45*	0.16	− *0.52*
CDR-IDS global score	n/a		0.10	− *0.46*

Statistically significant correlations (*p* < 0.05) are in italics

Older age was not significantly related to CDR-IDS domain, sum of boxes, or global decline scores. Excluding individuals with zero scores resulted in stronger relationships between older age and decline in memory (rho = 0.62, *p* = 0.14, *n* = 7), home and hobbies (rho = 0.50, *p* = 0.31, *n* = 6), CDR-IDS sum of boxes (rho = 0.32, *p* = 0.31, *n* = 12), and CDR-IDS global score (rho = 0.83, *p* < 0.05, *n* = 9). The sample size was *n* = 2 to 4 for the remaining four domains. Higher IQ was related to lower (better) cognitive function scores overall for both the CDR-QDS and CDR-IDS instruments with correlations (rho > − 0.45) reaching statistical significance.

### Relationship between CDR-QDS, CDR-IDS, and DSDS

Table 3 shows that decline in any one domain was significantly associated with decline in other domains and in summary scores within instrument (upper two panels) and across instruments (lower panel). The strongest relationship between the CDR-QDS and CDR-IDS was in the domain for personal care (rho = 0.87) and the weakest relationship was for judgment and decision-making (rho = 0.44) and orientation (rho = 0.45). There was a high correspondence between the CDR-QDS summary score and the CDR-IDS sum of boxes score (rho = 0.69).

**Table 3:** Spearman correlations across cognitive domains and summary scores within and between instruments

CDR-QDS (*n* = 34)	Memory	Orientation	Judgment	Community affairs	Home and hobbies	Personal care	Summary score
Orientation	*0.68*						
Judgment and problem solving	*0.69*	*0.85*					
Community affairs	*0.65*	*0.80*	*0.87*				
Home and hobbies	*0.65*	*0.62*	*0.85*	*0.81*			
Personal care	*0.69*	*0.68*	*0.90*	*0.87*	*1.00*		
Summary score	*0.91*	*0.70*	*0.81*	*0.80*	*0.75*	*0.77*	
CDR-IDS (*n* = 22)	Memory	Orientation	Judgment	Community affairs	Home and hobbies	Personal care	Sum of boxes
Orientation	*0.45*						
Judgment and problem solving	*0.54*	*0.59*					
Community affairs	*0.60*	*0.84*	*0.74*				
Home and hobbies	*0.43*	*0.48*	0.39	*0.64*			
Personal care	*0.58*	*0.59*	*0.50*	*0.74*	*0.62*		
Sum of boxes	*0.77*	*0.50*	*0.57*	*0.53*	*0.71*	*0.70*	
Global score	*0.87*	*0.60*	*0.48*	*0.57*	*0.47*	*0.72*	*0.87*
CDR-IDS	CDR-QDS						
	Memory	Orientation	Judgment	Community affairs	Home and hobbies	Personal care	Summary score
Memory	*0.66*	*0.62*	*0.48*	*0.60*	*0.45*	*0.43*	*0.51*
Orientation	*0.70*	*0.45*	*0.53*	*0.48*	*0.68*	*0.66*	*0.60*
Judgment and problem solving	*0.53*	0.35	*0.44*	0.40	*0.59*	*0.57*	0.41
Community affairs	*0.60*	*0.65*	*0.68*	*0.63*	*0.83*	*0.81*	*0.54*
Home and hobbies	0.42	*0.66*	*0.92*	*0.73*	*0.75*	*0.74*	*0.66*
Personal care	*0.52*	0.35	*0.66*	*0.63*	*0.87*	*0.87*	*0.63*
Sum of boxes	*0.61*	*0.51*	*0.69*	*0.54*	*0.61*	*0.61*	*0.69*
Global score	*0.69*	*0.48*	*0.53*	*0.51*	*0.60*	*0.59*	*0.66*

Statistically significant correlations (*p* < 0.05) are in italics

DSDS ratings resulted in 20 of 22 individuals having no dementia (data not shown). One person had a DSDS cognitive cutoff score of 2, a threshold for which 6-month reassessment is recommended [15]. One person had a DSDS rating consistent with early dementia (cognitive cutoff score ≥ 3 and total early and middle stage items present ≥ 10 but < 17). These two DS adults had, respectively, high summary CDR-QDS scores of 17 and 10, high CDR-IDS sum of boxes of 10.5 and 16, and the two highest global CDR-IDS scores of 2 and 3. Among the 20 DS adults rated as having no dementia according to the DSDS, 8 individuals had non-zero CDR-QDS scores (range 1–8) and 7 individuals had non-zero adjusted CDR-IDS global scores (0.5 and 1).

## Discussion

The modified CDR-QDS and CDR-IDS instruments captured a range of cognitive decline, provided complementary information and exhibited moderate to strong inter-correlations for the critical domains.

Our approach to account for premorbid function is similar to that of the CAMDEX-DS interview and DSQIID questionnaire. In the CAMDEX-DS, endorsement of functional difficulty is followed by a question of whether the difficulties represent deterioration [20]. In the DSQIID, accounting for premorbid function is implicit in the response options of individual items, which include the following: always been the case, always but worse, new symptom in past year, and does not apply [12]. The recommended cutoff score for the DSQIID is ≥ 20 for best sensitivity and specificity of the English version [12], and ≥ 22 for the Chinese version [21]. It was not possible to establish cutoff scores for the CDR-QDS because of our limited sample size.

In our study, accounting for premorbid abilities dramatically shifted the score distribution toward lower (no dementia) scores, which clearly demonstrates how not accounting for premorbid abilities would result in a high false positive rate of cognitive impairment. CDR scoring for the typical population emphasizes memory domain scores [22]. This emphasis was not uniformly seen in our sample. For example, unadjusted CDR-IDS scores showed that of 8 DS adults whose unadjusted memory domain scores were zero, 6 individuals had scores on other domains that suggested mild to severe dementia. CDR-IDS unadjusted scores across domains could include the entire scoring range from zero (no dementia) to three (severe dementia) for an individual. Such discrepancy in score magnitude across cognitive domains is not seen in typical aging adults (Dr. John Morris, personal communication, March 2018), and underscores both the wide individual variability in cognitive function in DS adults [34] and the importance of accounting for premorbid ability in dementia assessment. Adjusted CDR-IDS domain scores did not differ by more than one scoring category for any individual. It has been suggested that memory impairment may not be an early behavioral indicator of dementia in the DS population, rather emotional and behavioral problems (e.g., apathy, increased irritability, increased aggression, decreased interest in social interaction) and executive dysfunction (e.g., attention, planning) may be earlier symptoms of dementia in the DS population [12, 35–37], although these must be differentiated from treatable neuropsychiatric comorbidities in clinical practice. It has also been argued that memory problems have a similar time course of deterioration in DS as in the typical aging population. Administration of a cognitive battery to nearly 300 DS individuals aged 16 years and older showed that changes in performance in visuospatial associate memory, hand-eye coordination, and semantic verbal fluency preceded other cognitive changes, implicating these cognitive domains as early, prodromal behavioral markers of cognitive deterioration in DS [38]. In addition, change in tasks directly administered to DS individuals was seen earlier than change based on informant report, suggesting that the latter may not be sufficiently sensitive to detect early cognitive decline [38]. Similarly, the modified CDR-QDS and CDR-IDS instruments, entirely or mostly informant-based, may not be sensitive to detect subtle, early changes of cognitive decline, including memory problems.

Higher IQ was related to lower levels of cognitive decline. In the typically developing aging population, greater “cognitive reserve,” a measure that combines IQ, education, and lifelong engagement in cognitive activities [39], is also related to lower cognitive decline [40]. Here, we show a relationship in the lower distribution of IQ scores assessed using a single task.

Inclusion of the DSDS was meant to serve as an external validity index, but our sample did not reveal sufficient variability in DSDS scoring, and it is notable that a wider range of scores were elicited among the subjects using the CDR instruments. The vast majority of the sample was rated as having no dementia on the DSDS, but most had non-zero CDR-QDS and CDR-IDS scores; the 2 individuals flagged for reassessment or rated as early dementia on the DSDS had higher CDR-QDS scores, as well as CDR-IDS adjusted global scores consistent with moderate and severe dementia. The DSDS was developed and validated in a community sample of individuals the majority of whom had severe to profound intellectual disability which could result in low sensitivity of the DSDS in detecting dementia in higher functioning individuals [16, 17]. We note that in another instrument, the DSQIID, has been adapted by the National Task Group (NTG) on Intellectual Disabilities and Dementia Practices as an Early Detection Screen for Dementia (NTG-EDSD [41]), for wide use in dementia screening. Our results using comparable methods in the questionnaire version of the CDR support the utility of questionnaire-based screening, and future studies should continue to explore the utility of combining caregiver screening and direct observation in identifying dementia in DS and distinguishing it from other neuropsychiatric impairments that can result in cognitive decline in DS, most notably, catatonia, which is rare and controversial, but is treatable [42] and may assist clinicians in avoiding a false designation of dementia on the basis of observations or ratings that suggest cognitive decline, especially in younger adults with DS.

The participant interview portion of the CDR-IDS was difficult to administer to DS adults who were nonverbal or whose speech was limited or difficult to understand. The original CDR was developed for the typical population and in more extreme cases, it cannot capture the full range of disability. Ability to capture cognitive decline across the range of premorbid impairment in DS individuals, from low to high functioning, with a single instrument is a challenge [43]. In the CDR, “thinking and memory problems” are considered interchangeably, but caregiver informants regarded “thinking” and “memory” to be different phenomena and were often unsure how to answer questions that asked about thinking and memory problems together. Some spatial orientation questions were difficult because DS participants in our study were never alone and informants could not evaluate whether the DS adult would be able to find his/her way around familiar streets, for example. Ability to manage a household emergency like a small leak or fire did not seem applicable in our sample, again, since DS adults were almost never alone—even those who live independently have scheduled visitors and structured days. Ability to drive a vehicle or do calculations was absent in our entire sample.

Based on our data and on personal interaction with DS adults and their family members, ongoing research should continue to focus on elements of caregiver report and direct assessment of the DS individual that will optimize differential diagnosis. The primary goal of this study was to adapt the widely used CDR for assessment of dementia in adults with DS. We have shown that such adaptation was successful in capturing a range of cognitive decline and in shifting the distribution of decline scores toward lower/no impairment by adjusting original CDR questions to take into account premorbid intellectual and functional ability. Future work of the CDR-QDS and CDR-IDS should include comparison with existing instruments such as the CAMDEX-DS and DSQIID, with neuropsychological performance, and with other biomarkers, such as positron emission tomography for amyloid or tau.

## Conclusions

The modified CDR questionnaire (CDR-QDS) and interview (CDR-IDS) instruments capture a range of impairment in DS adults. Adjustment of cognitive decline scores for premorbid function dramatically shifted the score distribution toward no/low impairment and was an important element in identifying DS adults with suspicion for dementia. The DSDS indicated high suspicion of dementia in two of twenty-two individuals in this sample who had broadly elevated scores on the modified CDR instruments. The modified CDR instruments captured a range of subthreshold impairments in cognitive function that may prove extremely useful in prospective studies of the development of AD in DS. Moreover, future research is warranted to use quantitative scales such as these to help differentiate dementia from other neuropsychiatric problems in DS, including catatonia, adjustment disorder, and a range of psychiatric comorbidities, all of which are potentially treatable and for which inappropriate/premature assignment of a diagnosis of dementia may be extremely misleading and result in a major obstacle to appropriate care and treatment. For DS adults with elevated scores for cognitive impairment in this study, the impairment generally encompassed multiple cognitive domains and was not predominantly within the memory domain, as seen in the typical population.

## Data Availability

Data generated in this study are available from the corresponding author on reasonable request.

## Abbreviations

AD: Alzheimer disease
ADC: Alzheimer Disease Center
CAMDEX: Cambridge Examination for Mental Disorders in the Elderly
CDR: Clinical Dementia Rating Scale
CDR-IDS: CDR interview for Down syndrome
CDR-QDS: CDR questionnaire for Down syndrome
DLD: Dementia Questionnaire for People with Learning Disabilities
DMR: Dementia Questionnaire for Persons with Mental Retardation
DS: Down syndrome
DSDS: Dementia Scale for Down Syndrome
DSQIID: Dementia Screening Questionnaire for Individuals with Intellectual Disabilities
IQ: Intelligence quotient
NTG: National Task Group on Intellectual Disabilities and Dementia Practices
NTG-EDSD: National Task Group on Intellectual Disabilities and Dementia Practices Early Detection Screen for Dementia
SD: Standard deviation
